# A universal influenza virus vaccine candidate confers protection against pandemic H1N1 infection in preclinical ferret studies

**DOI:** 10.1038/s41541-017-0026-4

**Published:** 2017-09-14

**Authors:** Raffael Nachbagauer, Wen-Chun Liu, Angela Choi, Teddy John Wohlbold, Talia Atlas, Madhusudan Rajendran, Alicia Solórzano, Francesco Berlanda-Scorza, Adolfo García-Sastre, Peter Palese, Randy A. Albrecht, Florian Krammer

**Affiliations:** 1 Department of Microbiology, Icahn School of Medicine at Mount Sinai, New York, NY 10029 USA; 2 Graduate School of Biomedical Sciences, Icahn School of Medicine at Mount Sinai, New York, NY 10029 USA; 3 PATH US, Seattle, WA 98121 USA; 4 Global Health and Emerging Pathogens Institute, Icahn School of Medicine at Mount Sinai, New York, NY 10029 USA; 5 Department of Medicine, Icahn School of Medicine at Mount Sinai, New York, NY 10029 USA

## Abstract

Influenza viruses evade human adaptive immune responses due to continuing antigenic changes. This makes it necessary to re-formulate and re-administer current seasonal influenza vaccines on an annual basis. Our pan-influenza vaccination approach attempts to redirect antibody responses from the variable, immuno-dominant hemagglutinin head towards the conserved—but immuno-subdominant—hemagglutinin stalk. The strategy utilizes sequential immunization with chimeric hemagglutinin-based vaccines expressing exotic head domains, and a conserved hemagglutinin stalk. We compared a live-attenuated influenza virus prime followed by an inactivated split-virus boost to two doses of split-virus vaccines and assessed the impact of adjuvant on protection against challenge with pandemic H1N1 virus in ferrets. All tested immunization regimens successfully induced broadly cross-reactive antibody responses. The combined live-attenuated/split virus vaccination conferred superior protection against pandemic H1N1 infection compared to two doses of split-virus vaccination. Our data support advancement of this chimeric hemagglutinin-based vaccine approach to clinical trials in humans.

## Influenza: Defending against a common enemy

A vaccine against influenza targets non-varying parts of surface proteins to overcome the virus’ attempt at evading detection. Influenza viruses possess rapidly shifting surface proteins, effectively camouflaging themselves. These changes are making it difficult for vaccines to elicit reliable antibody responses against the threat. A team of researchers led by Florian Krammer and Randy A. Albrecht, of the United States’ Icahn School of Medicine at Mount Sinai, now describes a vaccine regimen that repeatedly targets a conserved component of the virus’ surface, prompting a broadly protective immune response. The conserved domains of the viral surface proteins are traditionally a more difficult target for vaccines as the immune systems of vaccinees have a preference for the varying domains. The team’s data, generated from ferret experiments, supports an investigation into the efficacy of this approach in humans.

## Introduction

Influenza virus infections cause significant morbidity and mortality worldwide every year.^1^ Currently licensed inactivated, recombinant, and live attenuated influenza virus vaccines (LAIVs) are proven to reduce clinical disease following influenza virus infection. However, influenza viruses are an evolving immunological target. The ever-changing nature of their surface glycoproteins, hemagglutinin (HA) and neuraminidase (NA), creates a difficult challenge for the production of effective vaccines. Seasonal influenza viruses, which cause annual epidemics, constantly escape from herd immunity by antigenic drift of their HA and NA proteins. Therefore, seasonal vaccine formulations have to be updated, re-formulated and re-administered on an annual basis.^2^ However, accurately predicting and selecting vaccine strains is difficult. Mismatches between vaccine strains and circulating strains cause a substantial decrease in vaccine efficacy.^3^ In addition, the specter of a new influenza pandemic resulting from the unpredictable emergence of a new antigenically shifted virus from an animal reservoir represents an unsurmountable challenge for current influenza vaccines. Production and distribution of matched pandemic vaccines takes approximately 6 months—an interval during which the human population is insufficiently protected from a pandemic influenza virus outbreak.^4^ The development of a pan-influenza vaccine that confers protection against homologous, drifted and shifted influenza virus strains would abolish the need for annual reformulation, and mitigate disease burden following the emergence of a pandemic influenza virus strain.

**Fig. 1:**
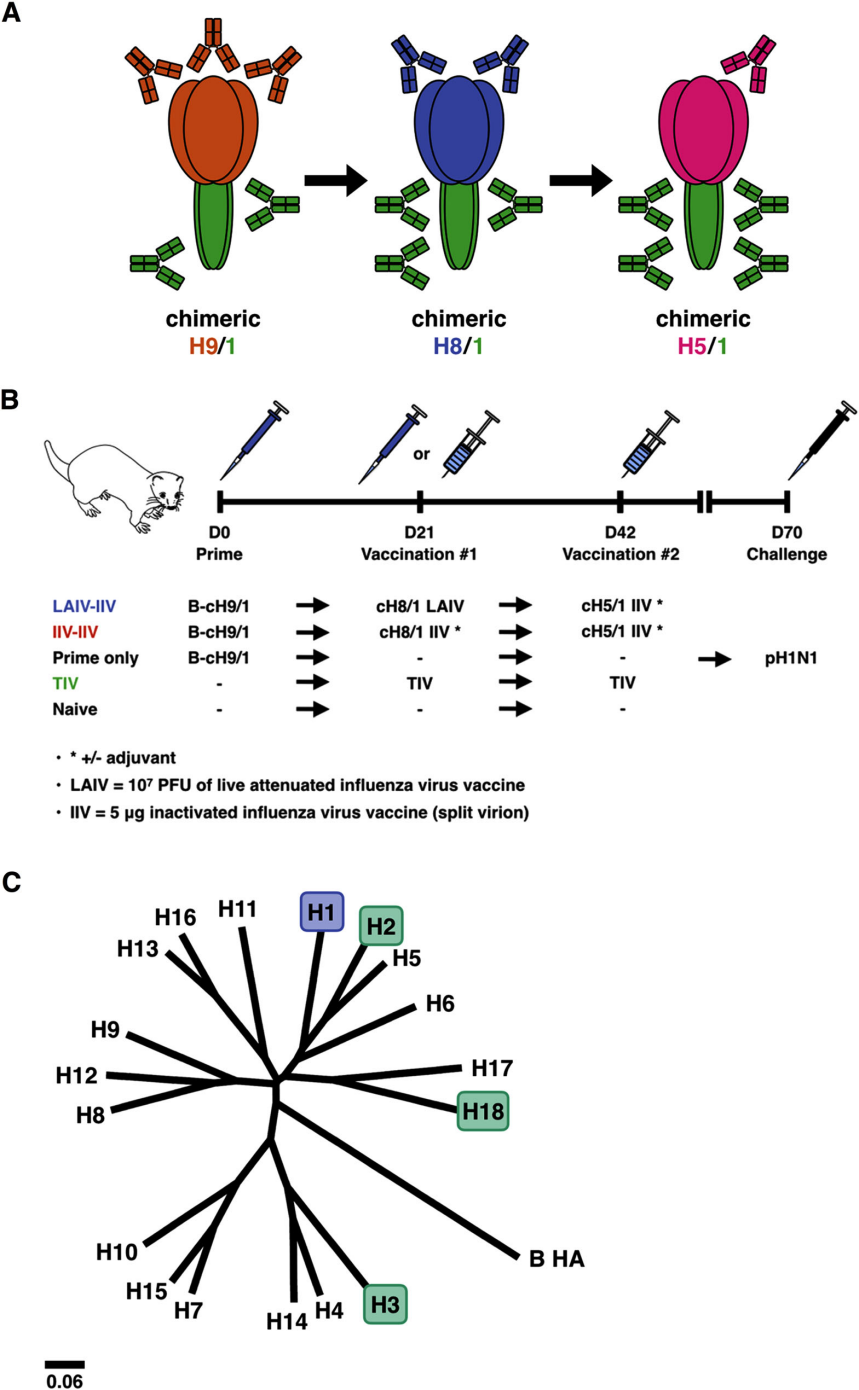
Vaccination overview and phylogenetic tree. **a** Chimeric HA vaccination. By exchanging the HA head domains, but retaining the same HA stalk domain, the antibody response can be redirected towards the otherwise immuno-subdominant stalk region. Repeated exposure to cHA constructs can boost HA stalk-specific antibodies to high titers. **b** Vaccination strategy. Ferrets in the cHA vaccination groups were primed with an influenza B virus expressing the cH9/1 HA, followed by cH8/1 vaccination either as LAIV or IIV (with or without adjuvant) on day 21. All cHA vaccination groups received a second boost on day 42 with cH5/1 IIV vaccine (with or without adjuvant). Ferrets that either received only the cH9/1 HA prime, received a seasonal trivalent influenza virus vaccine (TIV) twice or were naïve were included as control groups. All ferrets were challenged with pH1N1 virus on day 70. **c** Phylogenetic tree. All vaccines contained the HA stalk domain of H1 (highlighted in blue). ELISA serology was performed against H1 (highlighted in *blue*) as well as H2, H18 and H3 (highlighted in *green*). H2 is closely related to H1 HA, while H18 is the most distantly related HA within influenza A group 1. H3 is an influenza A group 2 HA and was included to measure cross-group reactive antibody titers. The scale bar (0.06) indicates the percent difference based on amino acid sequences

The discovery of broadly protective antibodies that bind to the conserved stalk domain of the HA and cross-react between HA subtypes sparked hopes for the development of a universal influenza virus vaccine.^5, 6^ Regular licensed influenza virus vaccines do not induce high antibody levels against the immuno-subdominant HA stalk domain but work through the induction of potent strain-specific antibodies against the immuno-dominant head domain.^7–11^ Sequential immunizations with chimeric HAs (cHAs), which consist of HA stalk domains derived from circulating influenza viruses in combination with ‘exotic’ head domains of influenza viruses that do not circulate in humans, can selectively boost anti-stalk antibodies.^12–16^ When the immune system is presented with a cHA, it responds mainly to the head domain but some priming effect against the stalk domain is achieved as well (Fig. 1a). Exposure to another cHA with the same stalk but a different head domain then boosts antibodies against the stalk domain but only induces a primary response against the novel head domain. This procedure can be repeated several times to induce high and protective titers against the conserved stalk domain. Importantly, adult humans are already primed against the stalk domain and it is expected that they mount a solid anti-stalk response after one to two vaccinations with cHAs.^10, 17, 18^ Another promising vaccination strategy that has recently been described includes heterologous prime boost strategies. In this case, an LAIV or a DNA vaccine is used as a priming immunization followed by a booster immunization with inactivated influenza virus vaccine (IIV) or protein vaccine.^19–23^ Here, using the ferret model, we compare a cHA-based LAIV prime—IIV boost immunization regimen with an IIV–IIV immunization regimen with and without the addition of the adjuvant AS03.

## Results

### Characterization of the cH8/1 LAIV strain

The objective of this study was the preclinical evaluation of a sequential immunization strategy with cHAs to induce HA stalk-specific immune responses that confer protection against influenza virus infection. The LAIV component, an A/Leningrad/134/17/57 master donor virus expressing the cH8/1 HA (H8 head domain on top of an H1 stalk domain) and the N1 NA from the 2009 H1N1 pandemic virus (cH8/1 LAIV), was rescued by reverse genetics under Good Laboratory Practices (GLP) at the US Centers for Disease Control and Prevention (CDC). Virus growth in eggs at permissive temperature led to titers above 10^8^ 50% egg infectious dose per ml. The temperature sensitive (*ts*) and cold-adapted (*ca*) phenotype of the cH8/1 LAIV was assessed in cell culture. The cH8/1 LAIV met the *ts* (reduction of 2 logs or more at 39 °C as compared to 33 °C) and *ca* (reduction of less than 2 logs at 25 °C as compared to 33 °C) criteria according to the criteria established by Jin and colleagues (Table 1).^24, 25^ Furthermore, the cH8/1 LAIV strain proved to be non-pathogenic in an intravenous pathogenicity test in chickens performed at the United States Department of Agriculture (USDA) according to the World Organization for Animal Health procedures for testing pathogenicity of influenza viruses.

**Table 1:** Characterization of the *ts* and *ca* phenotype of the cH8/1 LAIV

Virus titer (log_10_ TCID_50_/mL) at:						Phenotype	
Virus	39 °C	33 °C	25 °C	Δ33/39 °C	Δ33/25 °C	*ts* ^a^	*ca* ^b^
A/Puerto Rico/8/34	8.25	8.50	4.50	0.25	4.00	–	–
cH8/1 LAIV	0.50	5.50	5.00	5.00	0.50	+	+

^a^ defined as an at least 100-fold lower titer at 39 °C as compared to 33 °C

^b^ defined as less than an 100-fold lower titer at 25 °C as compared to 33 °C

**Fig. 2:**
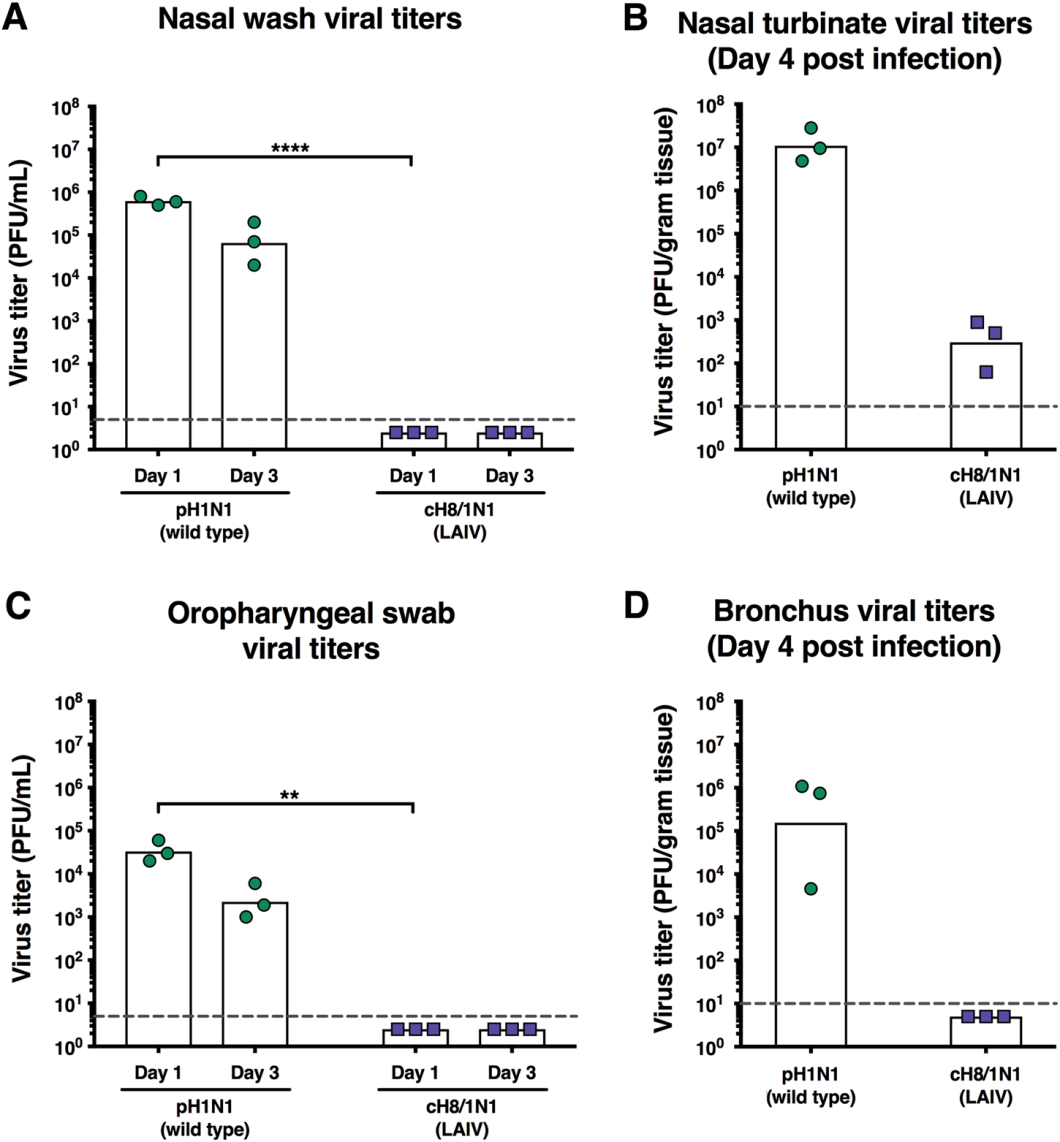
Replication of cH8/1N1 LAIV is attenuated in ferrets. Viral titers were measured by plaque assay. Animals infected with wild type pH1N1 (*n* = 3) are shown as *blue circles* and animals infected with cH8/1N1 LAIV (*n* = 3) are shown as *blue squares*. *White bars* indicate the GMTs of each group. The gray dashed line indicates the limit of detection. **a** Nasal wash viral titers. Viral titers in nasal washes were measured on day 1 and day 3 post infection. **b** Nasal turbinate viral titers. Viral titers in nasal turbinates were measured on day 4 post infection. **c** Oropharyngeal swab viral titers. Viral titers in oropharyngeal swabs were measured on day 1 and day 3 post infection. **d** Bronchus viral titers. Viral titers in the left medial bronchus of the upper lobe of the lung were measured on day 4 post infection. Groups in **a** and **c** were compared with a two-way ANOVA, followed by a Sidak’s multiple comparison test. The *asterisks* refer to the level of significance (zeros after the decimal point of the *p*-value): *P  ≤ 0.05; **P  ≤ 0.01; ***P  ≤ 0.001; ****P  ≤ 0.0001

The safety profile and replication phenotype was then assessed in 4-month-old neutered male ferrets. Following intranasal infection with an inoculation dose of 1 × 10^6^ PFU of cH8/1 LAIV, nasal wash and oropharyngeal swab samples were collected at days 1 and 3 post-immunization to quantify virus titers by plaque assay (Fig. 2). For comparison, ferrets were infected with a wild-type pandemic H1N1 virus isolate (pH1N1). Consistent with the anticipated attenuated phenotype, cH8/1 LAIV could not be detected by plaque assay in nasal wash and oropharyngeal swab samples, whereas the pH1N1 virus was present at high titers in recovered samples (Fig. 2a, c). Analysis of nasal turbinate samples collected at day 4 post immunization revealed that cH8/1 LAIV was present at a geometric mean titer (GMT) of 303.3 PFU/g tissue whereas the pH1N1 virus was present at a GMT of 1.1 × 10^7^ PFU/g tissue (Fig. 2b). Infectious cH8/1 LAIV could not be recovered from lung tissue; however, the control pH1N1 was detected at a GMT of 1.5 × 10^5^ PFU/g tissue (Fig. 2d). Collectively, the in vitro and in vivo data confirmed the expected attenuated phenotype of the cH8/1 LAIV strain.

### Immunization with cHA-based vaccine regimens induces stalk-reactive immunity in ferrets

Since adult humans have a primed repertoire of B-cells with specificities to the HA stalk domain, ^18, 26^ we used an experimental design that mimics this pre-existing immunity. Specifically, ferrets were initially primed with a recombinant influenza B virus expressing an influenza A virus cH9/1 HA. Infection with this virus primes the anti-stalk response without conferring any relevant (other than H9) HA head-based or internal protein-based protection from influenza A virus to the ferrets. Following the prime, animals were vaccinated with either the cH8/1 LAIV followed by adjuvanted or non-adjuvanted cH5/1 IIV or with adjuvanted or non-adjuvanted cH8/1 IIV followed by adjuvanted or non-adjuvanted cH5/1 IIV (Fig. 1b). Animals primed with B-cH9/1 and naive animals were used as control groups. Animals that received commercial Trivalent influenza virus vaccine (TIV) were added as ‘standard of care’ control. These animals were vaccinated with two doses of TIV without adjuvant which represents, according to CDC recommendations, the vaccination strategy for naive human subjects/children. Each group contained four ferrets.

**Fig. 3:**
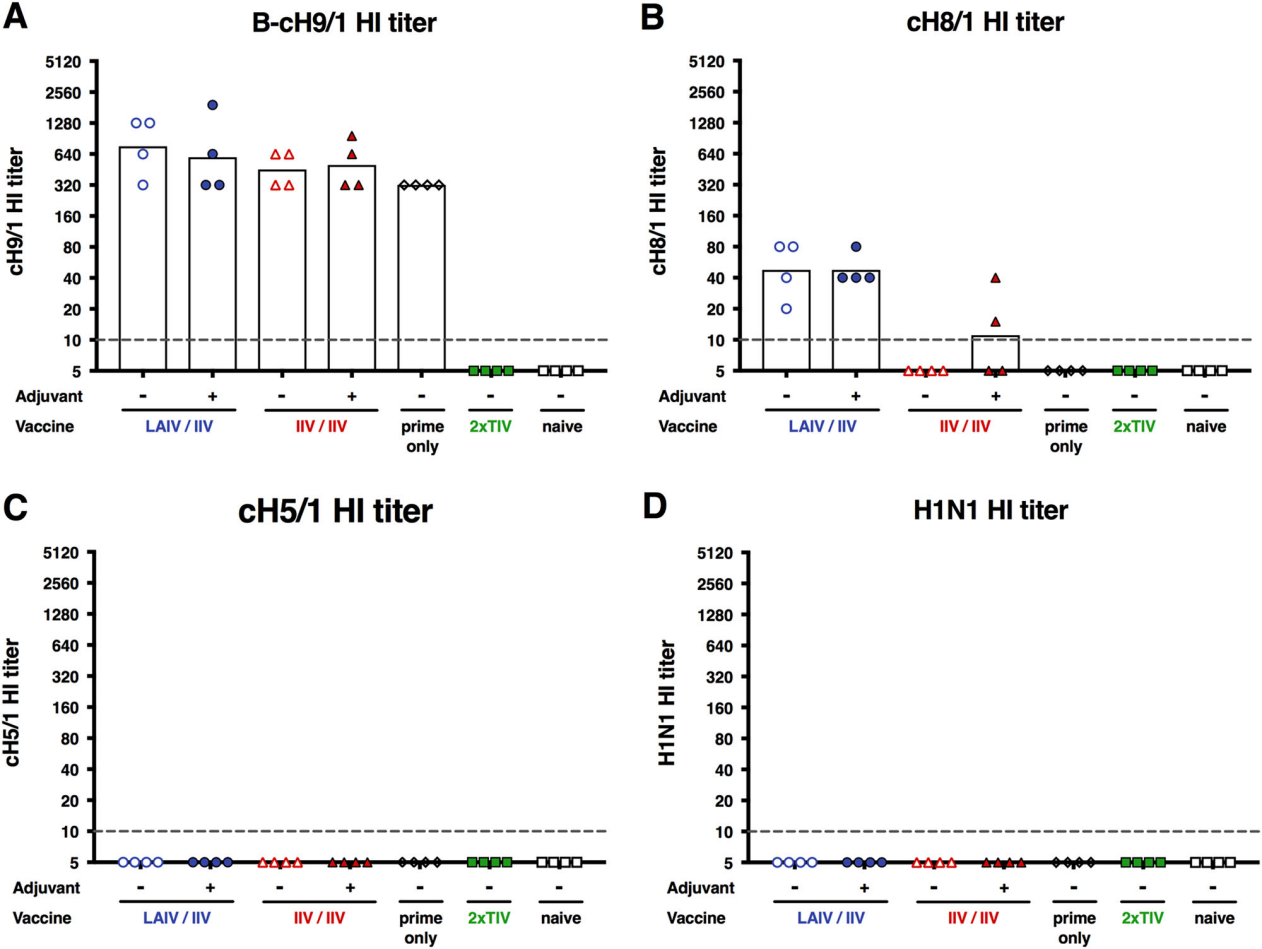
Hemagglutination inhibition titers. Hemagglutination inhibition titers are plotted on the *y*-axis. LAIV followed by IIV vaccinated animals are shown as *blue circles* (*empty circles*, without adjuvant; *full circles*, with adjuvant). IIV followed by IIV vaccinated animals are shown as *red triangles* (*empty triangles*, without adjuvant; *full triangles*, with adjuvant). Animals that received only the cH9/1 HA prime are shown as *white diamonds*. TIV-vaccinated animals are shown as *green squares* and naïve animals are shown as *white squares*. *White bars* indicate the GMTs of each group. Each point shows the titer for one animal (*n* = 4/group). The *gray dashed line* indicates the limit of detection. Titers were measured in pre-challenge sera (day 70). **a** B-cH9/1 hemagglutination inhibition titer. **b** cH8/1 hemagglutination inhibition titer. **c** cH5/1 hemagglutination inhibition titer. **d** pH1N1 hemagglutination inhibition titer

Hemagglutination inhibition (HI) titers were measured pre-challenge (day 70). As expected, ferrets immunized with the influenza B virus expressing cH9/1 HA seroconverted against H9 with HI titers of 320 to 1920 (Fig. 3a). Ferrets that received a booster immunization with the cH8/1 LAIV had detectable HI titers against H8 ranging from 20 to 80 (Fig. 3b). Of the two groups that received cH8/1 IIV, only two ferrets in the adjuvanted cH8/1 IIV group had detectable HI titers against H8. No ferrets seroconverted against H5 following the second booster immunization with cH5/1 IIV (Fig. 3c). Importantly, during the course of the immunization schedule, the ferrets remained HI naïve for pH1N1 (Fig. 3d).

**Fig. 4:**
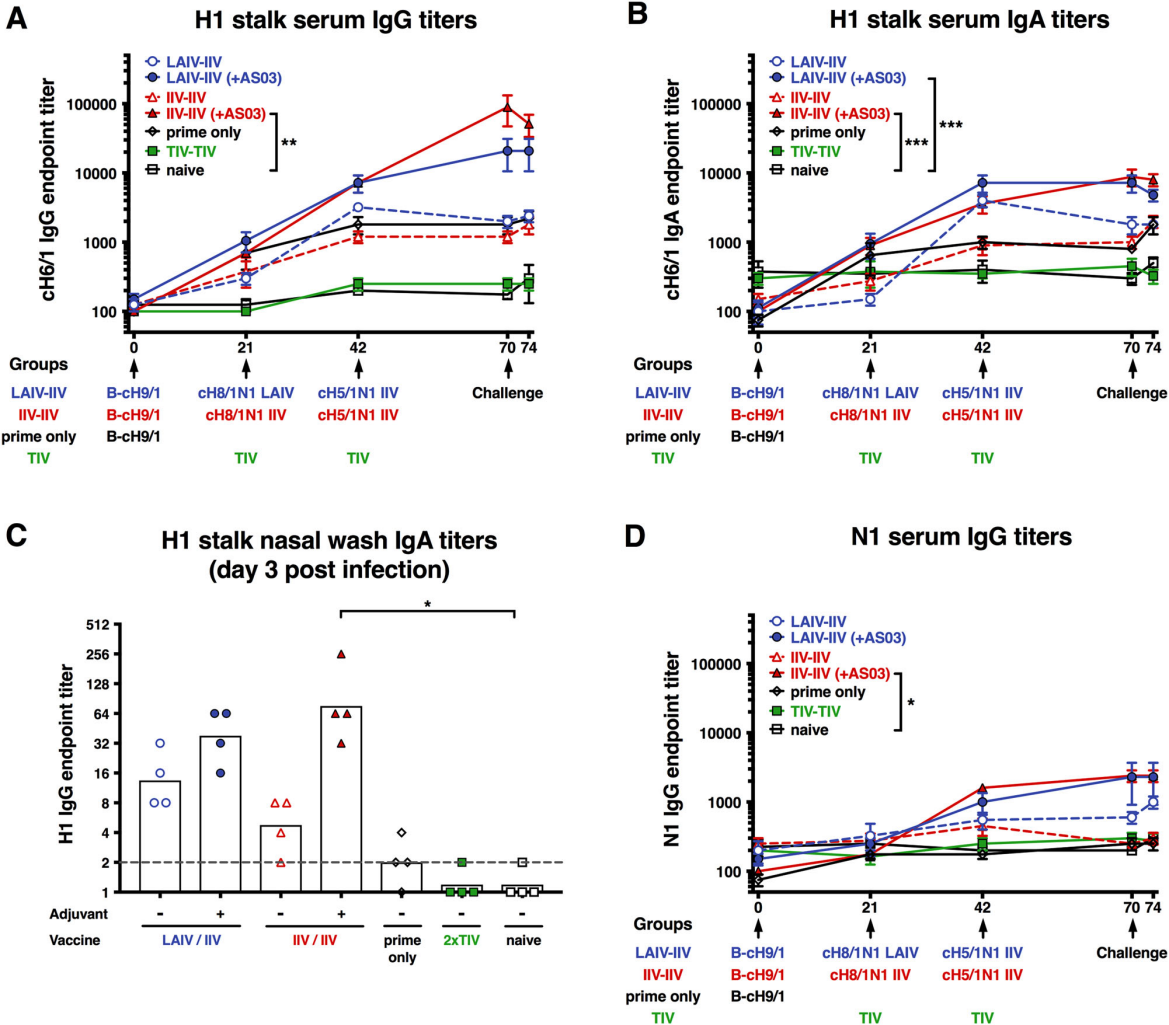
H1 and N1 specific antibody titers measured by ELISA. ELISA endpoint titers against cH6/1 protein are plotted on the *y*-axis. **a** H1 stalk serum IgG titers. The IgG antibody responses were measured on days 0, 21, 42, 70 and 74 (*x*-axis). Each point shows the mean titer and standard error of the mean for each group (*n* = 4/group). **b** H1 stalk serum IgA titers. The IgA antibody responses were measured on days 0, 21, 42, 70 and 74 (*x*-axis). Each point shows the mean titer and standard error of the mean for each group (*n* = 4/group). **c** H1 stalk nasal wash IgA titers. ELISA IgA titers were measured in nasal washes on day 3 post infection. Each point shows the titer for one animal (*n* = 4/group). White bars indicate the GMTs of each group. The *gray dashed line* indicates the limit of detection. **d** N1 serum IgG titers. The IgG antibody responses were measured on days 0, 21, 42, 70 and 74 (*x*-axis). Each point shows the mean titer and standard error of the mean for each group (*n* = 4/group). Vaccinated group means in **a**, **b** and **d** were compared to naïve animals with a two-way ANOVA followed by a Dunnett’s multiple comparison test (multiple time points) and a one-way ANOVA followed by a Dunnett’s multiple comparison test was used in **c** (single time point). The *asterisks* refer to the level of significance (zeros after the decimal point of the *p*-value): *P ≤ 0.05; **P ≤ 0.01; ***P ≤ 0.001; ****P ≤ 0.0001

B-cH9/1-primed animals induced an anti-stalk response that reached a plateau at a low titer (1:1800) at day 42 post prime as measured by enzyme-linked immunosorbent assay (ELISA, Fig. 4a). Animals that received non-adjuvanted LAIV–IIV and IIV–IIV vaccination regimens induced responses slightly higher (1:3200 for LAIV–IIV) or similar to the prime only animals with no apparent boost from the second vaccination (Fig. 4a). LAIV followed by adjuvanted IIV induced a strong anti-stalk response that was boosted by the second vaccination and peaked at a titer of 1:20800 on experimental day 70. The adjuvanted IIV–IIV regimen also induced a strong antibody response with a titer of 1:7200 after the first vaccination, which was boosted to 1:89600 after the second vaccination. Notably, the anti-stalk responses induced by the B-cH9/1 virus prime varied between the different vaccination groups (day 21). Naive animals and TIV (2xTIV) vaccinated animals did not induce a substantial IgG antibody response against the stalk domain during the study period (Fig. 4a). To corroborate titers measured with the cH6/1 HA substrate we also performed ELISA assays with H1-based headless HA consisting of only the stalk domain.^27^ Titers against headless HA were very similar to titers measured with cH6/1 HA, showing an overall good correlation (Supplementary Fig. 1).

In addition to the IgG anti-stalk response, we also monitored the serum IgA anti-stalk response (Fig. 4b). The serum IgA response followed a similar trend as the serum IgG response albeit at a lower magnitude. We also measured the anamnestic mucosal IgA anti-stalk response in day 3 post challenge nasal washes and found a similar pattern compared to serum IgA titers (Fig. 4c).

In addition, the serological IgG response to the N1 NA was tested (Fig. 4d). Only three groups induced notable N1 antibody levels. Vaccination with the non-adjuvanted LAIV–IIV strategy induced low anti-NA titers (1:600) that increased in an anamnestic response post challenge. A stronger response was induced when the adjuvanted LAIV–IIV regimen was used (1:2300 on day 70), which was similar to the observed response induced by the adjuvanted IIV–IIV regimen with a pre-challenge (day 70) peak titer of 1:2400.

**Fig. 5:**
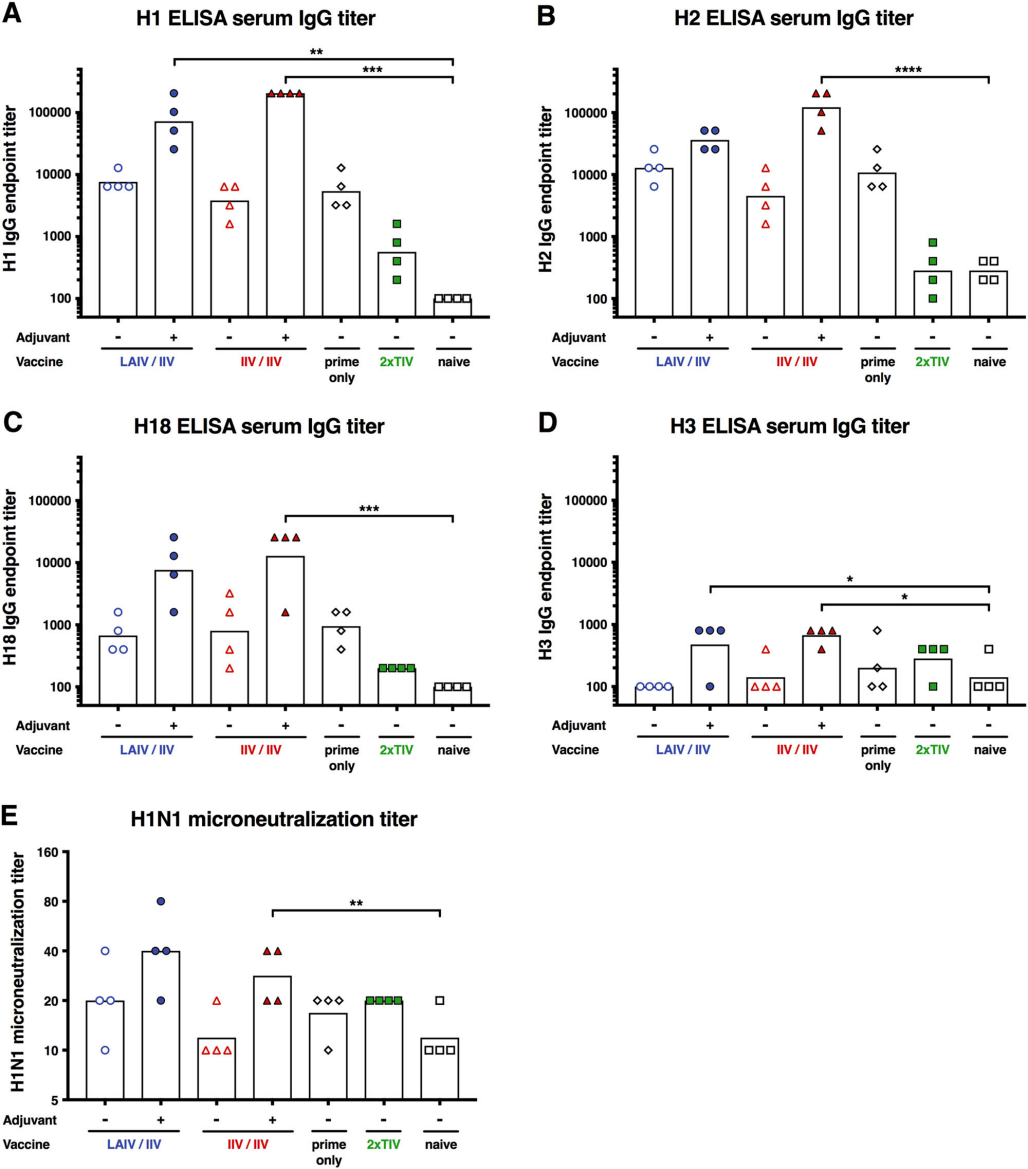
Antibody breadth measured by ELISA and neutralization. Titers are plotted on the *y*-axis. LAIV followed by IIV-vaccinated animals are shown as *blue circles* (*empty circles*, without adjuvant; *full circles*, with adjuvant). IIV followed by IIV-vaccinated animals are shown as *red triangles* (*empty triangles*, without adjuvant; *full triangles*, with adjuvant). Animals that received only the cH9/1 HA prime are shown as *white diamonds*. TIV-vaccinated animals are shown as *green squares* and naïve animals are shown as *white squares*. *White bars* indicate the GMTs of each group. Each point shows the titer for one animal (*n* = 4/group). The *gray dashed line* indicates the limit of detection. Titers were measured in pre-challenge sera (day 70). **a** H1 ELISA IgG titer. **b** H2 ELISA IgG titer. **c** H18 ELISA IgG titer. d H3 ELISA IgG titer. **e** pH1N1 neutralization titer. Groups were compared to naive animals with a one-way ANOVA followed by a Dunnett’s multiple comparison test. The asterisks refer to the level of significance (zeros after the decimal point of the *p*-value): *P  ≤ 0.05; **P  ≤ 0.01; ***P  ≤ 0.001; ****P  ≤ 0.0001

Furthermore, we analyzed the breadth of the antibody response induced by the cHA-based vaccination pre-challenge (day 70). Antibody titers in all groups were assessed against group 1 HAs H1, H2 and H18 as well as H3, a group 2 HA (Fig. 1c). Antibody responses to H1 and H2 were high in both the adjuvanted LAIV–IIV and IIV–IIV groups with the IIV–IIV groups reaching GMTs of 1:204800 and 1:121775, respectively and the LAIV–IIV group reaching GMTs of 1:72408 and 1:36204, respectively (Fig. 5a, b). Animals in the non-adjuvanted LAIV–IIV and IIV–IIV groups had lower titers than the adjuvanted groups but higher titers than the prime-only, 2xTIV and naive groups (Fig. 5a, b). Similar trends were observed for H18, a more distantly related group 1 HA (as compared to H1), but titers were lower in all vaccination groups. Antibody titers against H3—a group 2 HA—were very low but detectable, which could indicate low titers of cross-group reactive antibodies (Fig. 5d).^28^

Finally, we measured the neutralization titer against the pH1N1 challenge virus in a microneutralization assay (Fig. 5e). The titers were generally low with one animal in the non-adjuvanted LAIV–IIV group and two animals in the adjuvanted IIV group reaching a 1:40 titer. In the adjuvanted LAIV–IIV group two animals reached 1:40 and one animal reached 1:80. Titers in all other groups including the 2xTIV group had titers below 1:40. These results are consistent with earlier results with stalk-based vaccines in animal models in which low neutralization titers were measured.

### LAIV prime followed by IIV boost vaccination with cHA-immunogens confers superior protection compared to IIV prime followed by IIV boost and standard of care vaccination

**Fig. 6:**
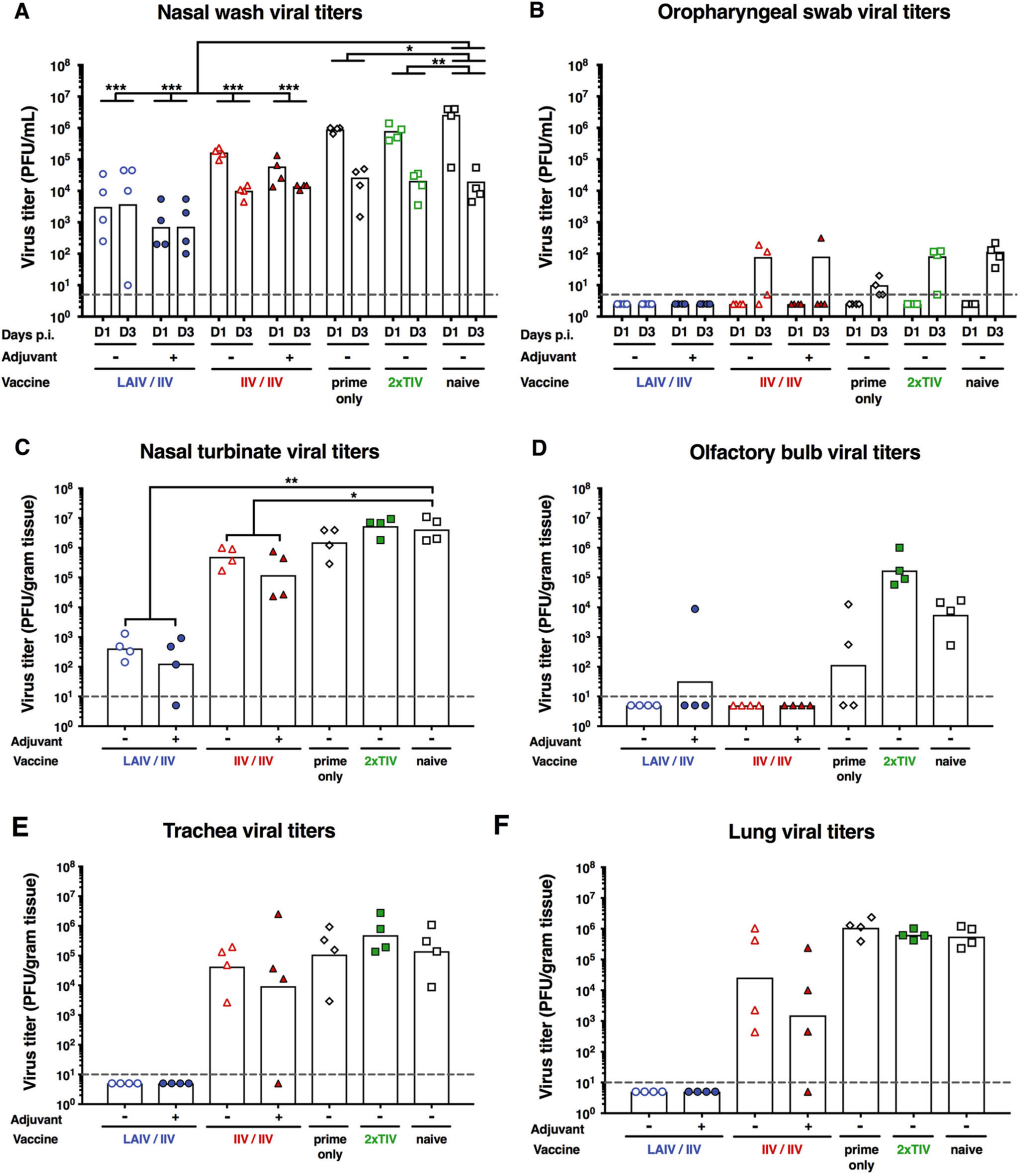
Virus titers post pH1N1 (10^4^ PFU) challenge. Viral titers were measured by plaque assay. LAIV followed by IIV-vaccinated animals are shown as *blue circles* (*empty circles*, without adjuvant; *full circles*, with adjuvant). IIV followed by IIV-vaccinated animals are shown as *red triangles* (*empty triangles*, without adjuvant; *full triangles*, with adjuvant). Animals that received only the cH9/1 HA prime are shown as white diamonds. TIV-vaccinated animals are shown as *green squares* and naïve animals are shown as *white squares*. *White bars* indicate the GMTs of each group. Each point shows the titer for one animal (*n* = 4/group). The *gray dashed line* indicates the limit of detection. **a** Nasal wash viral titers. Viral titers in nasal washes were measured on day 1 and day 3 post infection. **b** Oropharyngeal swab viral titers. Viral titers in oropharyngeal swabs were measured on day 1 and day 3 post infection. **c** Nasal turbinate viral titers. Viral titers in nasal turbinates were measured on day 4 post challenge. **d** Olfactory bulb viral titers. Viral titers in olfactory bulbs were measured on day 4 post challenge. **e** Trachea viral titers. Viral titers in the trachea were measured on day 4 post challenge. **f** Lung viral titers. Viral titers in the left medial bronchus of the upper lobe of the lung were measured on day 4 post challenge. Groups in **a** and **b** were compared to naïve animals with a two-way ANOVA (multiple time points), followed by a Dunnett’s multiple comparison test. Groups in **c**, **d**, **e** and **f** (single time point) were compared to naive animals with a one-way ANOVA followed by a Dunnett’s multiple comparison test. The asterisks refer to the level of significance (zeros after the decimal point of the *p*-value): *P  ≤ 0.05; **P  ≤ 0.01; ***P  ≤ 0.001; ****P  ≤ 0.0001

On day 70 post prime, all ferrets were challenged intranasally with 10^4^ PFU of a pH1N1 isolate. The challenge dose of 10^4^ PFU is the standard challenge dose established in the laboratory and was chosen here to make comparisons to earlier studies possible.^15, 16^ Nasal washes and oropharyngeal swab samples were obtained on day 1 and day 3 post challenge, and animals were euthanized on day 4 and the nasal turbinates, olfactory bulb, trachea, and lungs were harvested. This set-up is the standard challenge set-up and, as mentioned above, makes the data comparable to earlier studies. Naive animals showed geometric mean nasal wash titers in the 10^6^ and 10^4^ PFU/mL range on day 1 and day 3, respectively (Fig. 6a). Titers in the 2xTIV and prime-only groups were slightly lower than in the naive group. Animals vaccinated with the cHA-based IIV–IIV strategy showed a greater reduction of nasal wash titers, specifically on day 1 post challenge with a trend to lower titers in the adjuvanted versus the non-adjuvanted group. The lowest nasal wash titers on both day 1 and day 3 post-infection were found in the LAIV–IIV vaccinated groups with virus titer reductions of up to 3 logs as compared to naive animals. Again, the regimen with the adjuvanted IIV boost showed a trend towards lower titers than the group that received the non-adjuvanted boost. Oropharyngeal samples for all groups were negative on day 1 post infection and followed a similar trend as the nasal wash titers on day 3 post infection with undetectable titers in both LAIV–IIV groups (Fig. 6b).

Virus titers in the nasal turbinates—which usually support high levels of virus replication^16^—were between 10^6^ and 10^7^ PFU/gram tissue in the naive and TIV groups and slightly reduced in the B-cH9/1 group (10^6^ PFU/gram tissue range) (Fig. 6c). Animals that received the non-adjuvanted IIV–IIV and the adjuvanted IIV–IIV regimen had nasal turbinate titers in the range of 10^6^ and 10^5^ PFU/gram tissue respectively. Nasal turbinate titers in both LAIV–IIV groups were close to the limit of detection and one animal from the adjuvanted LAIV–IIV group had viral titers below the limit of detection. Virus replication in the olfactory bulb, which is an organ that is typically infected by pH1N1 isolates,^16^ was undetectable in all except for one cHA vaccinated animals (Fig. 6d).

We also assessed protection from virus replication in the lower respiratory tract. Interestingly, both LAIV–IIV vaccination regimens induced sterilizing immunity in the trachea with no detectable virus observed (Fig. 6e). The IIV–IIV vaccination and TIV vaccination groups showed tracheal titers that were very similar to the titers in naive ferrets. LAIV–IIV vaccination also completely blocked virus replication in the lung (Fig. 6f). Non-adjuvanted IIV–IIV vaccination caused a 2.5 log reduction of virus replication in the lungs compared to naive animals and adjuvanted IIV–IIV caused a reduction of approximately 3 logs. Again, no reduction was observed in the B-cH9/1 prime group or the TIV group.

**Fig. 7:**
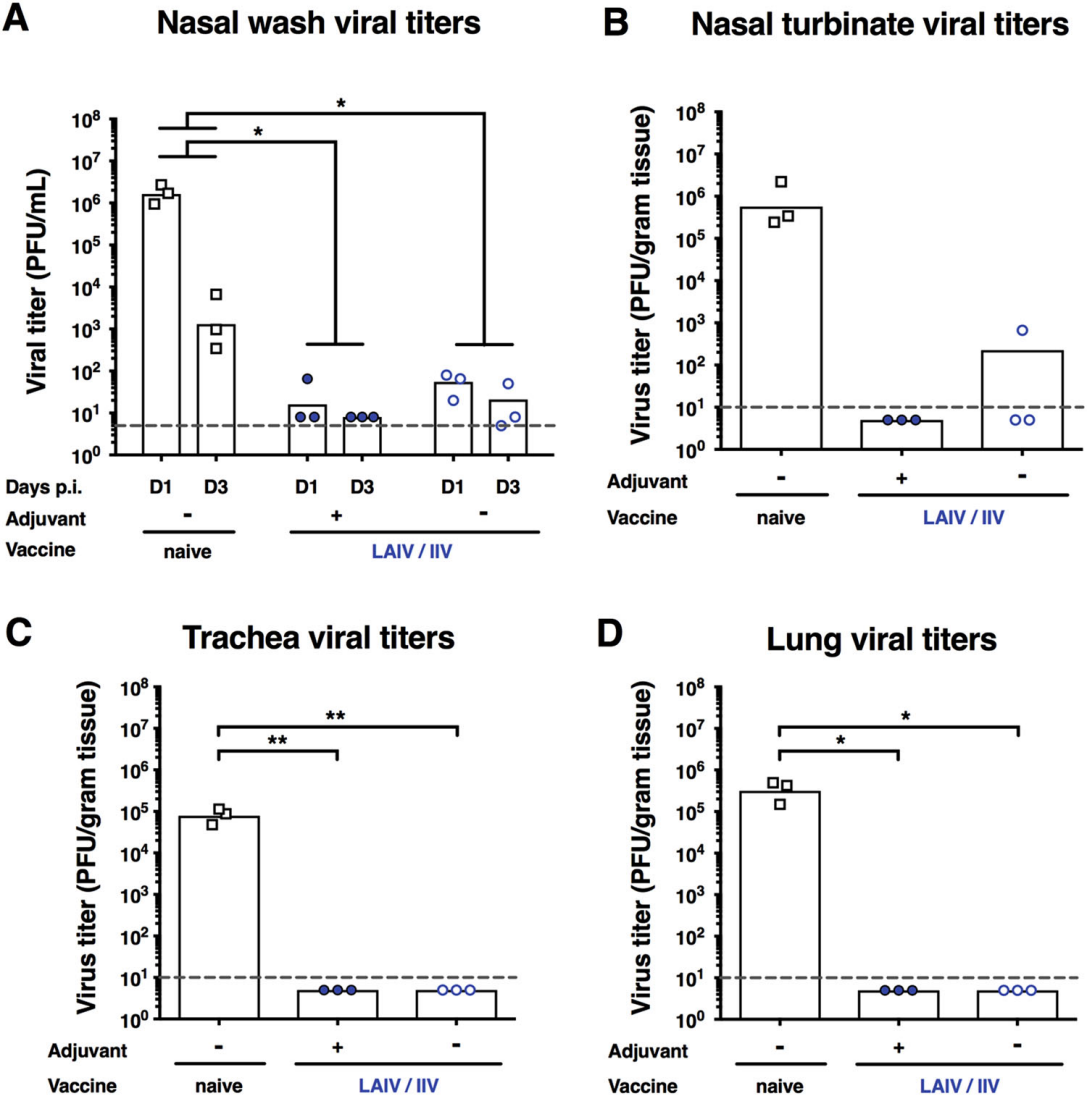
Virus titers post high dose pH1N1 (10^6^ PFU) challenge. Viral titers were measured by plaque assay. Naïve animals are shown as *white squares*, LAIV/IIV-vaccinated animals with adjuvant are shown *full blue circles* and LAIV/IIV-vaccinated animals are shown *empty blue circles* of the *x*-axis. *White bars* indicate the GMTs of each group. The *gray line* indicates the limit of detection. **a** Nasal wash viral titers. Viral titers in nasal washes were measured on day 1 and day 3 post infection. **b** Nasal turbinate viral titers. Viral titers in nasal turbinates were measured on day 4 post challenge. **c** Trachea viral titers. Viral titers in the trachea were measured on day 4 post challenge. **d** Lung viral titers. Viral titers in the left medial bronchus of the upper lobe of the lung were measured on day 4 post challenge. Groups in a were compared to naïve animals with a two-way ANOVA (multiple time points), followed by a Dunnett’s multiple comparison test. Groups in **b**, **c** and **d** (single time point) were compared to naive animals with a one-way ANOVA followed by a Dunnett’s multiple comparison test. The asterisks refer to the level of significance (zeros after the decimal point of the *p*-value): *P ≤ 0.05; **P ≤ 0.01; ***P ≤ 0.001; ****P ≤ 0.0001

Finally, to test if the use of adjuvant is necessary in this influenza vaccination model the LAIV/IIV vaccination strategies with and without AS03 adjuvanted boost were repeated followed by a higher pH1N1 challenge dose (10^6^ PFU) with the goal to detect differences in protection, specifically in the lower respiratory tract. The nasal wash and nasal turbinate virus titers were low in both groups, which indicated good protection even at a higher challenge dose (Fig. 7a, b). No significant differences could be detected between the groups, but more ferrets in the unadjuvanted group had detectable virus titers. Both groups were again completely protected from viral replication in the lower respiratory tract (Fig. 7c, d). While the benefit of adding adjuvant to the LAIV–IIV vaccination regimen is clear when antibody titers are assessed, we were unfortunately not able to draw conclusions based on protection from infection, even with the higher 10^6^ PFU challenge dose.

## Discussion

The aim of this preclinical study was to combine LAIV and IIV vaccination approaches to induce broad immune responses against the influenza virus HA, specifically against the conserved stalk domain. In recent years, heterologous prime-boost regimens (e.g., LAIV followed by IIV or DNA vaccines followed by protein vaccines) have been demonstrated to induce strong and broad antibody responses post-boost despite almost undetectable immune responses post-prime. While the mechanism of this phenomenon is still not well understood, its efficiency has been demonstrated in animal models and humans.^19–23, 29–31^ A second technology—based on cHAs that can be used in sequential vaccination regimens to specifically boost antibody responses to the conserved HA stalk domain—has been successfully tested in several animal models as well.^13–16, 32^ In this study, we tested if a combination of these two technologies would further enhance and broaden the immune response against the influenza virus surface glycoproteins. Furthermore, we assessed if adding AS03 as adjuvant to the IIV component of the vaccine regimen would be beneficial.

The heterologous LAIV–IIV vaccination strategy clearly outperformed the homologous IIV–IIV vaccination regimen in terms of protection both in the upper and in the lower respiratory tract. However, it is important to mention that the IIV–IIV regimens tested also afforded significant protection as compared to the current standard of care (2xTIV in naive individuals). Addition of the adjuvant certainly had a substantial effect on antibody titers against both the HA stalk (serum IgG and IgA) and against the NA. The contribution of the adjuvant to protection was less pronounced but a trend could be observed both for the LAIV–IIV and the IIV–IIV regimens in most tissues. One limitation was that animals vaccinated with the LAIV–IIV regimen (adjuvanted and non-adjuvanted) had no detectable virus in lungs, trachea, olfactory bulb and oropharyngeal samples, and it is therefore hard to make a valid comparison. A follow-up experiment with a high dose virus challenge again showed complete protection of the lower respiratory tract, but revealed a similar trend of slightly higher viral titers in the unadjuvanted vaccine group in the upper respiratory tract.

Another important observation is that there is a disconnect between anti-stalk titers measured in serum and the observed protection. As an example, the non-adjuvanted LAIV–IIV regimen greatly outperformed the adjuvanted IIV–IIV regimen in terms of protection despite a 32-fold lower anti-stalk titer induced by LAIV–IIV (non-adjuvanted) pre-challenge as compared to IIV–IIV (adjuvanted). Several factors could account for this disconnect. Antibodies against the N1 NA were induced by the adjuvanted IIV–IIV but also by the adjuvanted and nonadjuvanted LAIV–IIV regimen. While these titers were low it has been shown that anti-NA antibodies can protect from viral challenge in mouse and ferret models.^33–35^ LAIV is administered mucosally and is therefore thought to induce strong local immune response in tissue-resident immune cells. This could lead to an increased secretion of protective anti-stalk and anti-NA IgA. While we did not find higher mucosal IgA titers in the post-challenge nasal washes of non-adjuvanted LAIV–IIV as compared to the adjuvanted IIV–IIV vaccinated animals, it is unclear if this anamnestic response would correlate with the pre-challenge mucosal concentration of anti-stalk secretory IgA titers. Furthermore, LAIV replicates actively in the upper respiratory tract of vaccinees, leading to intracellular antigen expression, which primes for strong adaptive cellular immune responses. This cell-based immune response could very well account for the observed difference in protection. Finally, replicating LAIV viruses induce unspecific innate immune responses. It has been recently shown that such innate immune responses can actually lead to unspecific protection mediated by Club cells—an effect that can last up to 12 weeks in mice.^36^

The tested vaccination strategy elicited potent antibody responses against influenza A group 1 HAs, but low antibody responses against H3, a group 2 HA. A complete pan-influenza virus vaccine will need to consist of a trivalent formulation that contains cHAs for group 2^14^ and B influenza viruses. Such a multivalent vaccine could induce stalk-reactive antibody responses against all influenza subtypes.

All vaccines and adjuvants used in the study are of high translational potential. The LAIV component used in this study grows to high titers in eggs, which allows for large-scale manufacturing. The backbone of the LAIV is based on the Leningrad vaccine strain, which has been licensed and successfully used in humans.^37^ The split virus vaccines were produced in a cell-culture-based approach that has previously been used to manufacture pandemic H5N1 split virus vaccines by GlaxoSmithKline.^38^ Furthermore, the adjuvant AS03 has been licensed for use in humans for pandemic influenza virus vaccines.^39^

In summary, this study demonstrates that both cHA-based heterologous LAIV–IIV and homologous IIV–IIV vaccination strategies induce broadly reactive anti-stalk antibodies and protect ferrets from challenge with pH1N1 virus with the heterologous vaccination strategy outperforming the homologous vaccination regimen in terms of protection. While we chose to test the vaccines in primed ferrets that better mimic the immune status of adult humans, we hypothesize that the LAIV prime—which also mimics natural infection—might be the better choice for vaccination of children who lack pre-existing anti-stalk immunity. The data resulting from this study warrants testing of this cHA-based heterologous prime boost regimen as a universal influenza candidate in clinical trials.

## Materials and methods

### Cells, viruses, proteins and vaccines

Madin–Darby canine kidney (MDCK) cells were maintained in complete Dulbecco’s modified Eagle medium (Life Technologies) supplemented with antibiotics (100 U/mL penicillin- 100 μg/mL streptomycin [Pen-Strep]; Gibco) and 10% fetal bovine serum (HyClone). Virus stocks were grown in 8–10 day old specific pathogen-free embryonated chicken eggs (Charles River Laboratories) for 2 days at 37 °C or for 3 days at 33 °C. Eggs were then chilled to 4 °C overnight, allantoic fluid was harvested, cleared using low speed centrifugation, aliquoted and quantified by plaque assay on monolayers of MDCK cells in the presence of tosyl phenylalanyl chloromethyl ketone (TPCK)-treated trypsin. Plaques were visualized by immunostaining or crystal violet staining.

Recombinant proteins including cH6/1 (containing the H6 HA head domain from A/mallard/Sweden/81/02 and the H1 stalk domain from A/Puerto Rico/08/34), H1 (from A/California/04/09), H2 (from A/mallard/Netherlands/5/99), H3 (from A/Hong Kong/4801/14), H18 (from A/bat/Peru/33/10), H1 based headless HA (from A/Puerto Rico/8/34^27^) and N1 (from A/California/04/09) were expressed in High Five cells grown in serum free SFX medium (HyClone) using the baculovirus expression system.^34, 40, 41^

IIV split vaccines were produced in EB66 cells based on cH5/1_Cal09_N1_Cal09_ (cH5/1 IIV) and cH8/1_Cal09_N1_Cal09_ (cH8/1 IIV) viruses and were provided by GlaxoSmithKline.^38^ The cH5/1_Cal09_N1_Cal09_virus expresses an HA head domain of A/Vietnam/1203/04 (H5N1), an H1 stalk domain and N1 NA from A/California/04/09 virus while the internal genes were derived from A/Puerto Rico/8/34 virus.^12, 32, 42^ The cH8/1_Cal09_N1_Cal09_ expresses an HA head domain from A/mallard/Sweden/24/02 (H8N4) while the remaining genes as well as the stalk domain are the same as for the cH5/1_Cal09_N1_Cal09_ virus.^12, 32^ The IIV vaccines were administered as non-adjuvanted (5 μg HA antigen in 500 μl of phosphate-buffered saline pH7.4 (PBS)) or adjuvanted with Adjuvant System 03 (AS03; 5 μg HA antigen in 0.25 mL of PBS plus 0.25 mL of AS03^43, 44^).

The cH8/1 LAIV expressed the same cH8/1_Cal09_ and N1_Cal09_ as the cH8/1 IIV but was based on the A/Leningrad/134/17/57 live-attenuated backbone which features a *ts* and *ca* phenotype.^45^ The cH8/1 LAIV seed virus was rescued at the Center for Disease Control (CDC) under GLP conditions.^46^ pDZ plasmids for the virus rescue were cloned at the Icahn School of Medicine at Mount Sinai. GLP-grade PB2, PB1, PA, NP, M and NS segments of A/Leningrad/134/17/57 virus were synthesized (DNA2.0, Newark, CA) from sequences deposited in the Global Initiative for Sharing Influenza Data (GISAID) online database (EPI555079 to EPI555086).

The B-cH9/1 is based on a recombinant B/Yamagata/16/88 backbone with an HA that features the HA stalk domain from H1N1 strain A/Puerto Rico/8/34 and the head domain from H9N2 strain A/guinea fowl/Hong Kong/WF10/99. The HA segment was constructed as described by Hai and colleagues^47^ and was first described in.^13^

### Characterization of the cH8/1 LAIV

Upon rescue, the cH8/1 LAIV was expanded in specific pathogen-free embryonated eggs (Charles River Laboratories) at the permissive temperature of 33 °C for 72 h. Stocks were prepared, quantified by plaque assay and sequence verified by Sanger sequencing. The replicative phenotype was assessed in MDCK cells following a well-established TCID_50_ protocol.^25^ Briefly, confluent layers of MDCK cells in 96-well plates were infected with serial 1/2-log dilutions of the cH8/1 LAIV and A/Puerto Rico/8/34 H1N1 (as non-*ts*, non-*ca* control) in minimal essential medium (MEM) containing TPCK-treated trypsin and Pen-Strep. The plates were incubated at 39 °C, 33 °C and 25 °C and the TCID_50_ dose was calculated according to the Spearman & Kaerber algorithm based on cytopathogenic effect.^48^ Per definition of Jin and colleagues^24^ a *ts* phenotype is defined by a titer at 39 °C that is at least 100-fold lower than at 33 °C and a *ca* phenotype is defined by a titer at 25 °C being less than 100-fold lower than at 33 °C. Furthermore, the cH8/1 LAIV virus was sent to the USDA where an in vivo intravenous pathotyping experiment in chickens was performed according to the World Organization for Animal Health procedures for testing the pathogenicity of influenza viruses. Finally, we examined the attenuation phenotype of cH8/1 LAIV by intranasal infection of ferrets with an inoculation dose of 1 × 10^6^ PFU. A group of ferrets inoculated with wild-type A/California/04/09 virus served as a control. For this experiment 4-month-old neutered male Fitch ferrets (*n* = 3) were confirmed to be seronegative for circulating H1N1, H3N2, and B influenza viruses prior to purchase from Triple F Farms (Sayre, PA). All animal procedures performed in this study were performed in accordance with the Institutional Animal Care and Use Committee (IACUC) guidelines and Public Health Service Policy on Humane Care and Use of Laboratory Animals. The animal protocol was reviewed and approved by the IACUC of the Icahn School of Medicine at Mount Sinai.

### Ferret immunization and challenge

Three to 5-month-old spayed female Fitch ferrets were confirmed to be seronegative for circulating H1N1, H3N2, and B influenza viruses prior to purchase from Marshall BioResources (North Rose, NY). All animal experiments were performed according to a protocol approved by the IACUC of the Icahn School of Medicine at Mount Sinai. The sample size was chosen according to the standards in the field. The space requirements and cost associated with the ferret model did not allow for a larger sample size. Ferrets randomly assigned to different treatments by assigning a treatment to a group of four animals. Figure 1b summarizes the prime/boost immunizations administered to each immunization group (*n* = 4) of ferrets. The universal vaccination regimen is based on boosting pre-existing HA-stalk antibodies which are present at low levels in human adults.^18^ To mimic this scenario, naive ferrets in the chimeric vaccination groups were primed by intranasal infection with an inoculation dose of 1 × 10^6^ PFU (in 1 mL of PBS) of influenza B virus expressing cH9/1 HA.^13^ This priming method prevents the establishment of immunity against influenza A virus internal proteins and does not interfere with a later pH1N1 virus challenge. Importantly, while animals were assigned to groups prior to priming, their immune status post-priming was not assessed until after the challenge, meaning that the animals were de facto randomized after the prime. Three weeks later, two groups of primed ferrets (LAIV–IIV + and LAIV–IIV− groups, + /− indicates the presence of adjuvant in the IIV boost) were intranasally vaccinated with a 0.5 mL dose containing 1 × 10^7^ PFU of cH8/1N1 LAIV, followed 3 weeks later by intramuscular vaccination with cH5/1N1 IIV (5ug/HA in 0.25 mL of PBS). The IIV vaccine doses for ferrets in the LAIV–IIV + group were adjuvanted with 0.25 mL of Adjuvant System 03 (AS03), while the vaccine doses for the ferrets in the LAIV–IIV− group were mixed with 0.25 mL of PBS (0.5 mL final injection volume). To compare the LAIV–IIV approach to an exclusive IIV–IIV approach, two groups of ferrets (IIV–IIV + and IIV–IIV−, + /− indicates the presence of adjuvant in prime and boost) received cH8/1N1 IIV followed by cH5/1N1 IIV intramuscularly (5ug/HA in 0.25 mL of PBS per dose) with the same vaccination interval. Ferrets in the IIV–IIV + group received both doses of AS03 adjuvanted vaccine (0.25 mL) and ferrets in the IIV–IIV− group received both doses unadjuvanted vaccine (0.25 mL of PBS). An unvaccinated group served as a negative control to compare the protection conferred by cHA vaccination with naïve animals (naive). To show any effects of the cH9/1 B virus prime on viral loads, a prime only group was included (prime-only). Finally, a “standard of care” group of ferrets received 2 doses of a human dose TIV containing a pH1N1 component on days 21 and 42 of the experiment (2xTIV; 2015–2016 formulation of the seasonal influenza vaccine, Fluvirin, Seqirus). Four weeks after the last booster immunization, all animals were challenged intranasally with an inoculation dose of 10^4^ PFU of A/California/4/09 in 1 mL of PBS. An inoculation dose of 10^6^ PFU (high dose challenge) was used in a smaller follow up experiment. Nasal washes and oropharyngeal swabs were collected from anesthetized ferrets on days 1 and 3 after challenge. On day 4 after challenge, the animals were euthanized by intracardiac injection of Sleepaway euthanasia solution (Fort Dodge, IA) and tissues (olfactory bulbs, nasal turbinates, trachea, and lung) were collected from each individual ferret to quantify viral titers by plaque assays. Serum was collected at baseline, day 21, day 42, day 70 and on the last day of the virus challenge experiment.

### Enzyme-linked immunosorbent assay (ELISA)

ELISAs were performed with recombinantly produced proteins to measure HA-specific antibodies in serum and nasal washes. Furthermore, antibodies against N1 were measured in serum by ELISA. To measure serum IgG, 96-well plates were coated with 50 µl of 2 µg/mL of antigen in sodium bicarbonate buffer (pH 9.4) per well and incubated overnight at 4 °C. Plates were washed three times with PBS containing 0.1% Tween 20 (PBS-T) and blocked with 220 µl of blocking solution (PBS-T containing 3% goat serum (Gibco) and 0.5% milk powder) for 1 h at room temperature. After initial blocking, the volume was discarded and 100 µl of blocking solution was added to each well. An additional 90 µl of blocking solution was added to the first well of the dilution series. Ten microliters of serum pre-diluted 1:5 in PBS were then added to the first well of the dilution series (resulting in a 1:100 starting dilution). Samples were 2-fold serially diluted by transferring 100 µl to the next well. Two columns were left blank on each plate. Plates were incubated for 2 h at room temperature, washed three times with PBS-T and 50 µl of horse radish peroxidase-labeled anti-ferret IgG (Alpha Diagnostic International, #70530) at a concentration of 1:5000 was added to each well. Plates were incubated for 1 h at room temperature, washed four times with PBS-T and developed by adding 100 µl of SigmaFast OPD per well. After 10 min, the reaction was stopped by addition of 3 M hydrochloric acid and plates were read at a wavelength of 490 nm in a plate reader (Biotek Synergy H1). The average plus three standard deviations of blank wells were calculated as a cut-off value. If a sample did not dilute out below the cut-off, it was re-run at a higher starting dilution. The same procedure was used to measure IgA titers in nasal washes and serum. However, a starting dilution of 1:2 was used for the nasal washes (100 µl of nasal wash + 100 µl of blocking solution in the first well). Horseradish peroxidase-labeled anti ferret IgA antibody (Novus Biologicals, NBP1-73728) was used at a 1:3000 concentration as secondary antibody.

### Receptor-destroying enzyme (RDE) treatment of sera

For neutralization and HI assays, sera were treated with RDE of *Vibrio cholerae* (Denka Seiken, Chuo-ku, Tokyo, Japan) according to manufacturer’s instructions. RDE-treated serum samples were then treated with three volumes (based on original serum volume) of 2.5% sodium citrate solution and then incubated at 56 °C for 30 min. Three volumes of PBS (based on original serum volume) were added to each sample for a final dilution of 1:10.

### Hemagglutination inhibition (HAI) assays

HAI assays are performed as described elsewhere.^49^ Working stocks for each influenza virus strain were prepared by diluting the virus stock to a final HA titer of 8 HA units/50 µL. Two-fold dilutions (25 µL) of the RDE-treated sera in PBS prepared in 96 V-well microtiter plates were then mixed with 25 µL of the working stock of each influenza virus strain (each well contains a final volume of 50 µL). The serum virus samples were then incubated at room temperature for 30 min to allow any HA-specific antibodies present in the serum to neutralize the influenza virus. To each well, 50 µL of a 0.5% suspension of chicken red blood cells was then added, and the assays were then incubated at 4 °C until the red blood cells in the PBS control sample formed a button. Samples were tested in technical duplicates. The HI titer was defined as the reciprocal of the highest dilution of serum that inhibited red blood cell hemagglutination by influenza virus and the mean titers of duplicates were reported.^16^

### Microneutralization assays

Two-fold dilutions (50 µL) of the RDE-treated sera in sterile Opti-MEM (Invitrogen, Carlsbad, CA) were mixed with 200 PFU of influenza virus (5 µL). The serum virus samples were then incubated at room temperature for 60 min to allow any HA-specific antibodies present in the serum to neutralize the influenza virus. The serum-virus samples (55 µL) were then transferred to MDCK cells cultured in 96-well flat bottom plates. Following virus adsorption for 60 min, the serum-virus inocula were removed, and the MDCK cells were cultured for 4 days in Opti-MEM supplemented with 1 µg/mL of TPCK-trypsin (Sigma-Aldrich). Virus production was determined by HA assay. The neutralization titer was defined as the reciprocal of the highest dilution of serum that neutralizes 200 PFU of influenza virus. Samples were tested in technical duplicates and the mean titers were reported.

### Statistical analysis

For comparisons of two groups with a single time point an unpaired t-test followed with a Welch’s correction for unequal standard deviations was used. For comparisons of two groups with multiple time points a two-way analysis of variance (ANOVA) followed by a Sidak’s post-test to adjust for multiple comparisons was performed. For comparisons of more than two groups with a single time point, column means were compared to naive animals with a one-way ANOVA followed by a Dunnett’s post-test to adjust for multiple comparisons. For comparisons of more than two groups with multiple time points, column means were compared to naive animals with a two-way ANOVA followed by a Dunnett’s post-test to adjust for multiple comparisons. All statistical analyzes were performed in Graphpad Prism 7. The asterisks shown in the figures refer to the level of significance (zeros after the decimal point of the *p*-value): *P  ≤ 0.05; **P  ≤ 0.01; ***P  ≤ 0.001; ****P  ≤ 0.0001. No samples or animals were excluded from the analysis. Serum analysis and viral titer quantification were performed after completion of the vaccination/challenge experiment. Serological assays and viral titration was done in an unblinded manner.

### Data availability

The data that support the findings of this study are available from the corresponding author upon request.

## Electronic supplementary material

Supplementary Figure 1
