# Supplementary material for: A universal influenza virus vaccine candidate confers protection against pandemic H1N1 infection in preclinical ferret studies
Source: NPJ Vaccines. 2017 Sep 14;2:26. doi: 10.1038/s41541-017-0026-4 (PMC5627297; doi:10.1038/s41541-017-0026-4)
Supplement: Supplementary file 1 — Supplementary Figure 1 [file 41541_2017_26_MOESM1_ESM.docx]

**
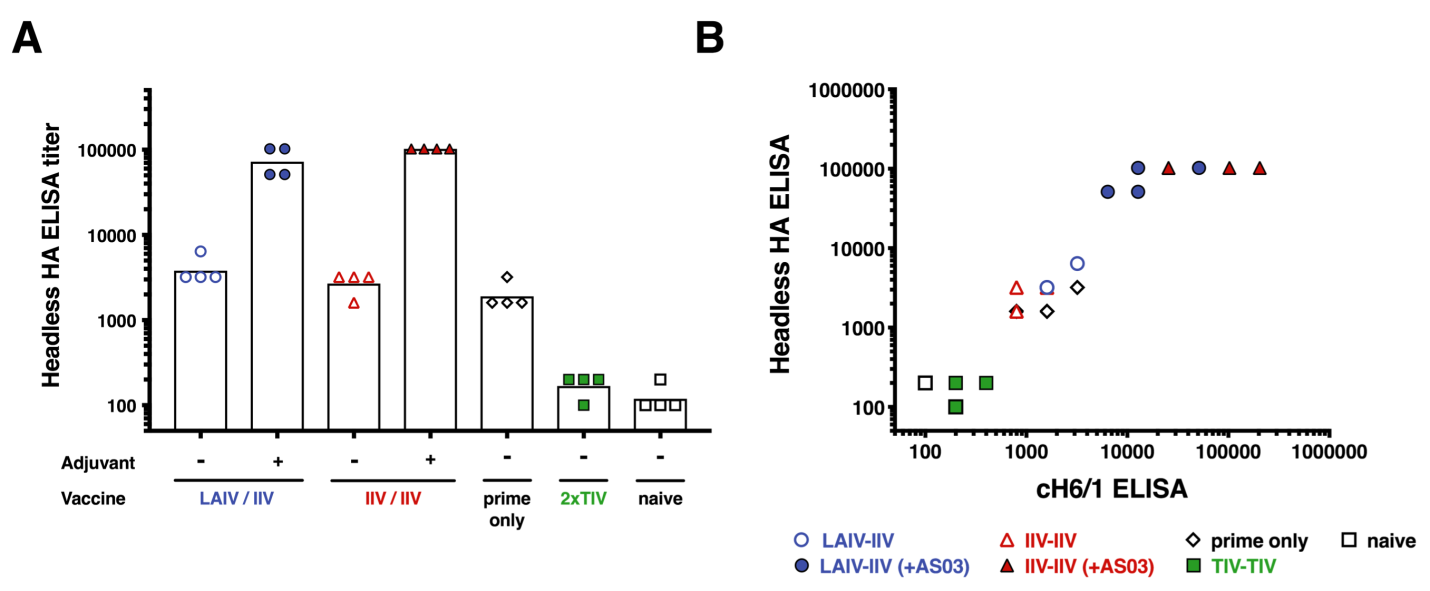
Supplementary Figure 1: HA stalk-specific antibodies measured by ELISA with an H1 based headless HA construct.**

**A) Headless HA ELISA titers.** ELISA IgG endpoint titers against a headless HA protein are plotted on the y-axis. White bars indicate the GMTs of each group. Each point shows the titer for one animal (n=4/group). **B) Correlation of headless HA and cH6/1 ELISA titers.** ELISA IgG endpoint titers measured against headless HA are plotted on the y-axis and ELISA IgG endpoint titers measured against cH6/1 are plotted on the x-axis. Each point shows the corresponding titers for one animal (n=4/group).
